# Environmental Effects on Public Health: An Economic Perspective

**DOI:** 10.3390/ijerph6082160

**Published:** 2009-07-31

**Authors:** Kyriaki Remoundou, Phoebe Koundouri

**Affiliations:** Department of International and European Economic Studies, Athens University of Economics and Business, 76, Patission Street, Athens 104 34, Greece; E-Mail: kremoundou@aueb.gr

**Keywords:** health risks, environmental management, valuation

## Abstract

In this article we critically review the economic literature on the effects of environmental changes on public health, in both the developed and the developing world. We first focus on the economic methodologies that are available for the evaluation of the effects (social costs and benefits) of environmental changes (degradation/preservation) on public health. Then, we explain how the monetary valuations of these effects can feed back in the construction of economic policy for creating agent-specific incentives for more efficient public health management, which is also equitable and environmentally sustainable. Our exposition is accompanied by a synthesis of the available quantitative empirical results.

Every minute, five children in developing countries die from malaria or diarrhoea. Every hour, 100 children die as a result of exposure to indoor smoke from solid fuels. Every day, nearly 1,800 people in developing cities die as a result of exposure to urban air pollution. Every month, nearly 19,000 people in developing countries die from unintentional poisonings.

Source: Health and Environment: Tools for Effective Decision-Making: the WHO/UNEP Health and Environment Linkages Initiative Review of Initial Findings, 2004.

## 1. Introduction

The environment affects our health in a variety of ways. The interaction between human health and the environment has been extensively studied and environmental risks have been proven to significantly impact human health, either directly by exposing people to harmful agents, or indirectly, by disrupting life-sustaining ecosystems [1]. Although the exact contribution of environmental factors to the development of death and disease cannot be precisely determined, the World Health Organization (WHO) has estimated that thirteen million deaths annually are attributable to preventable environmental causes [1]. The report also estimates that 24% of the global disease burden (healthy life years lost) and 23% of all deaths (premature mortality) are attributable to environmental factors, with the environmental burden of diseases being 15 times higher in developing countries than in developed countries, due to differences in exposure to environmental risks and access to health care.

However, huge economic development and population growth result in continuing environmental degradation. Intensification of agriculture, industrialization and increasing energy use are the most severe driving forces of environmental health problems. For countries in the early stages of development the major environmental hazards to health are associated with widespread poverty and severe lack of public infrastructure, such as access to drinking water, sanitation, and lack of health care as well as emerging problems of industrial pollution. However, environmental health hazards are not limited to the developing world. Although at a lesser extend, environmental risks are also present in wealthier countries and are primarily attributed to urban air and water pollution. Occurrences of Asthma are rising dramatically throughout the developed countries, and environmental factors appear to be at least partly to blame [1]. The Millennium Ecosystem Assessment [2] synthesis report warns that the erosion of ecosystems could lead to an increase in existing diseases such as malaria and cholera, as well as a rising risk of new diseases emerging.

Climate change is also posing risks to human population health and well-being and thus is emerging as a serious concern worldwide [3–6]. In 2000 climate change was estimated to be responsible for approximately 2.4% of worldwide diarrhoea and 6% of malaria [1]. According to the IPCC third assessment report the world temperature is expected to further rise during the century, implying increased health threats for human populations, especially in low-income countries. Reviewing the US literature addressing health impacts of climate variability and change Ebi *et al.* [7], conclude that climate change is expected to increase morbidity and mortality risks from climate-sensitive health outcomes such as extreme heat events, floods, droughts and fires. A spread in vector–borne diseases, like malaria, is also expected [8,9]. A study in Mexico revealed that lower greenhouse gases emissions would result in avoidance of some 64,000 premature deaths over a twenty year period [4]. Leading death causes worldwide (2004) are summarized in Table 1.

This paper provides a review of the literature on valuation studies eliciting monetary values associated with reduced environmental risk and in particular focusing on reduced indoor and outdoor air pollution, enhanced water quality and climate change mitigation. The findings of the valuation studies have important policy implications, since the environmental risk factors that are studied can largely be avoided by efficient and sustainable policy interventions. Minimizing exposure to environmental risk factors by enhancing air quality and access to improved sources of drinking and bathing water, sanitation and clean energy is found to be associated with significant health benefits and can contribute significantly to the achievement of the Millennium Development Goals of environmental sustainability, health and development.

## 2. Economic Valuation Techniques

Quantifying the impacts of environmental degradation on human health is essential for the development of well-informed policies by the health sector and consequently many valuation studies have been conducted worldwide the past decades addressing environmental risks to public health. The main approaches for health impact valuations can be broadly classified into revealed and stated preference techniques. The first take into account observable market information which can be adjusted and used for revealing an individual’s valuation. Revealed preferences include cost of illness, human capital surveys, hedonic pricing and the Quality Adjusted Life Year studies. In stated preferences studies the market for the good is ‘constructed’ through the use of questionnaires. The two most-well-known stated preference methods are the Contingent Valuation Method (CVM) and the Choice Experiments (CE).

Cost of illness studies measure the direct (medical costs, nursing care, drugs) and indirect (opportunity) economic costs associated with a disease and estimate the potential savings from the eradication of the disease. Human capital surveys estimate the productivity loss measured in workdays due to illness. This approach also values loss of life based on the foregone earnings associated with premature mortality. The notion is that people should be willing to pay at least as much as the value of the income they would lose by dying prematurely.

Damage costs estimates from environmental hazards for the economy as a whole are also obtained through general equilibrium macroeconomic modeling. These studies assess welfare impacts in a national or international level by examining all the sectors of the economy and estimating environmental health impacts on parameters of the economy like income and consumption.

The Quality Adjusted Life Year (QALY) studies measure both the quality and quantity of life. The values for a Life Year range from 0, implying death, to 1, implying a year of perfect health. Therefore, QALYs provide an indication of the benefits from a healthcare intervention in terms of health-related quality. Combined with the costs of providing different interventions, a cost-effectiveness analysis (cost per QALY) can follow to allow for comparisons of different interventions. A monetary value can also be placed on a QALY to estimate the dollar benefits of a health intervention or policy and allow for a subsequent cost-benefit analysis. Stated Willingness to Pay, elicited through a contingent valuation study or a discrete choice study, is often used, to monetize QALYs. Other methods to value a QALY include time-trade-offs, standard gamble and the visual analogue scale. Hedonic pricing methods assess differences in the price of housing in polluted or unpolluted areas, or the difference in wages between hazardous and non-hazardous jobs. Variations in housing prices and wages reflect the value of health damages avoided to those individuals and therefore reveal individual’s willingness to pay to avoid damages.

Stated preference approaches include the Contingent Valuation Method and Choice Experiments. The respective differences between the two methodologies relate to the way in which the economic values are elicited. In a contingent valuation questionnaire respondents are presented with a valuation scenario that describes the changes in the provision of the public good resulting from the policy under evaluation and, in the simplest open-ended format, are asked about their maximum Willingness to Pay for the policy to be implemented. Grounded on Lancaster’s theory of value [11], choice experiments describe the good under evaluation in terms of its characteristics, attributes, and the levels these attributes take. One of the attributes is usually price, so that the marginal value of the other attributes can be evaluated in monetary terms. Accordingly, respondents are presented with a set of alternatives constructed from different combinations of the levels of attributes, and are asked to choose their most preferred. Similarly a choice experiment can be used to examine policy implications of a policy or management strategy with policy impacts being the attributes to be valued.

Before valuing the health damage the establishment of a dose-response function relating pollutant concentrations to health impacts is required [12]. The impacts of environmental degradation on mortality, expressed as the increase in the probability of premature death, and quality of life, expressed as reduction of the morbidity risk, are thus initially considered. Accordingly respondents are asked to either state their willingness to pay for a prevention scenario (stated preference approach) or the benefits are elicited through the costs that would be saved if the risk was eradicated (cost of illness studies). Benefits are mainly reported by calculating the Value of a Statistical Life (For a review of the literature calculating the value of a statistical life based on labor and housing market data see Viscusi and Aldy [13].). The Value of Statistical Life (VSL) is calculated by dividing the value of a small risk change by the actual change in risk and thus captures the effect of small changes in the risk of premature death for a large population of potentially exposed people [14].

Since primary data collection to establish the dose response functions or proceed with the valuations can be expensive and time-demanding, there is substantial policy interest in using benefit transfer techniques. In this context, original values from existing studies are transferred to policy sites after correcting for certain parameters. Given the number of valuation studies, several meta-analyses studies have been recently conducted. Following this approach valuation estimates from existing studies are collected and the determinants of these estimates are examined. In a meta-analysis regression, therefore, the dependent variable is a common summary statistic, such as a predicted variable for the Willingness to Pay, whereas the independent variables include characteristics of the primary data, study design, valuation method, sample size, model specification, econometric methods, date of publication [15]. Meta-analyses can feedback the establishment of value transfer functions to estimate values for policy sites of interest based on properly adjusted information from existing studies on similar sites, study sites [16].

Each of the methods described has its own strengths and limitations. The choice between these methods should be case-study driven, that is, it should be a function of case-study-specific data availability and socio-economic-political framework. In human capital surveys it is often difficult to assign wages for housework or non-cash labour. Hedonic methods require a well functioning market for housing or labour, which internalizes the health risks associated with a location or a job. The cost of illness approach fails to capture the full damage of illness, such as psychological suffering and physical pain and should be thus treated as a lower bound of the total value of health risks aversion [17]. Using QALY to estimate the damage costs may also lead to underestimations [18]. Opponents of QALYs use argue that these measures cannot in general appropriately represent individual preferences for health, while they are consistent with the utility theory under very restrictive conditions [19]. QALYs finally ignore the distributional effects arising from the dependence of WTP on income. Macroeconomic modelling is often based on simplistic assumption regarding the economy while many impacts are unquantifiable and are thus not modelled [5].

The contingent valuation method (CVM), although widely used, has been criticised for its lack of reliability since it is associated with biases, such as hypothetical bias, strategic bias, yes-saying bias and embedding effect [20,21]. Hypothetical bias contends that respondents may be prepared to reveal their true values but are not capable of knowing these values without participating in a market in the first place. Strategic bias occurs when respondents deliberately under- or overstate their WTP. Respondents may understate their WTP if they believe that the actual fees they will pay for provision of the environmental resources will be influenced by their response to the CV question. Conversely, realising that payments expressed in a CV exercise are purely hypothetical, respondents may overstate their true WTP in the hope that this may increase the likelihood of a policy being accepted. Yea-saying bias indicates that respondents may express a positive WTP because they feel good about the act of giving for a social good although they believe that the good itself is unimportant while embedding bias implies that WTP is not affected by the scale of the good being offered. To address these, the Blue Ribbon Panel under the auspices of NOAA [22] has made recommendations regarding best practice guidelines for the design and implementation of contingent valuation studies.

Comparing the stated preference methods for environmental valuation Boxall *et al.* [23] argue that choice experiments (CEs) have important advantages over others valuation methods mainly because of their experimental nature which enables the representation of different states of the environment using attributes and levels of specific choice situations. The latter has a clear benefit compared to other valuation methods as it leads respondents to explicitly make trade-offs between the various attributes of the situation and thus provides policy-makers with valuable information about public preferences for many states of the environment. Environmental health effects of a policy or project can therefore be explicitly addressed and valued. Both CVM and CEs studies represent preferences that are consistent with utility theory, with CEs being also able to solve for some of the biases present in the CVM. Therefore it is our opinion that the application of CEs should be further enhanced in health economics to evaluate health impacts of environmental policies.

## 3. Economic Assessment of Environmental Health Impacts: Empirical Evidence

There is increasing recognition that linked environment and health impacts require economic assessment in order to receive adequate consideration in policy [1]. Consequently, a huge increase in the number of valuation studies trying to quantify the environmental impacts on human health in monetary terms and elicit public preferences for health and environmental policies that reduce the risk of illness or mortality has been experienced in recent years.

In the subsequent sections important applications of the valuation techniques that have been conducted to estimate social benefits associated with increased air and water quality as well as climate change aversion are reviewed. Limitations of the existing research are addressed in the concluding section and directions for future work are suggested. For quick reference a table summarizing each study’s main features (that is author, case study country, environmental hazard and valuation result) can be found in the Appendix. All valuations have been converted to 2006 euros (2006 average $0.797 = 1 euro).

### 3.1. Air Quality

Air pollution is a major environmental risk to health and is estimated to cause approximately two million premature deaths worldwide per year [24]. A reduction of air pollution is expected to reduce the global burden of disease from respiratory infections, heart disease, and lung cancer. As air quality is a major concern for both developed and developing countries, a large number of empirical studies attempting to monetize the benefits to health generated by improved air quality have appeared in the literature worldwide.

Pearce [12] provides a summary of the main studies conducted to that day valuing health damages from air pollution in the developing world. In particular, valuation estimates for health symptoms and risks of mortality attributable to particulate matter, lead, nitrogen and sulphur oxides and low level ozone are reported. The main conclusion from the literature review is that some forms of air pollution, notably inhalable particulate matter and ambient lead, are serious matters for concern in the developing world since they are associated with severe health damages in monetary terms.

Since then a number of valuation studies have been conducted in developing countries estimating social benefits from air pollution reduction in terms of either averted mortality or averted morbidity due to air pollution mitigation strategies. To provide economic estimations of health risk reductions authors rely on existing epidemiological studies that establish the relationship between pollution concentrations and health hazards. Valuation studies are then conducted to monetize health outcomes given the number of exposures and the associated risk predicted from the dose-response functions.

In the literature addressing air pollution in both developed and developing world, contingent valuation studies are mainly implemented. The health consequences from alternative pollution abatement policies are explicitly stated in the valuation scenario and respondents are asked their maximum willingness to pay to contribute in the implementation costs of the policy under evaluation.

Mortality and mobility effects of air pollution have been studied through contingent valuation in the developing world [25–28]. To provide economic grounds for supporting investment in air pollution abatement a cost benefit-analysis is often applied [29–31]. Results from valuation studies adopting a benefit transfer framework to circumvent the time and money demands of conducting an original study are also reported in the literature [32,33]. A cost of illness approach is employed by Gupta [34] to estimate the monetary benefits to individuals from health damages avoidance due to air pollution reduction in India. Health costs are considered to be incurred due to adverse effects of air pollution on health i.e., the loss in wages due to workdays lost from work and expenditures on mitigating activities. While the majority of studies addressed outdoor air pollution, Chau *et al.* [35] combine revealed and stated preference techniques to estimate the monetary benefit gains from improved indoor air quality. Authors conduct a meta-analysis to estimate the concentration-response coefficients for different health outcomes to which they then assigned economic value based on existing values from the literature. Findings indicate that there would be some benefit gains for the owners-employers and the society if certain regular filter sets were adopted. The amount of benefit gains by the owners-employers increases with the average salary level of employees and duration that they stay in offices.

Hedonic studies have been also applied to estimate a relationship between housing prices and housing attributes, including health risks associated with air pollution. The value people place on reduced health risks through improved air quality are inferred by their willingness to pay more for houses with better air quality, all else being equal. Delucchi *et al.* [36] provide a meta analysis of hedonic pricing studies addressing health risks from air pollution. Comparing results with studies applying the damage function approach, authors find evidence that hedonic price analysis does not capture all of the health costs of air pollution because individuals are not fully informed about all of the health effects to incorporate them into property values.

According to the authors’ knowledge, in developed countries environmental health studies are limited and all consist of contingent valuation studies in Europe. To assess morbidity risk reduction benefits, Navrud [37] conduct a contingent valuation study to estimate the willingness-to-pay (WTP) to avoid additional days of seven light health symptoms (coughing, sinus congestion, throat congestion, eye irritation, and headache, shortness of breath and acute bronchitis) and asthma. Mean WTP for an environmental program that would result to reduced health risks (avoiding one additional day of the health symptoms) ranges from 16.62 euros for coughing to 44.2 for the shortness of breath. Mortality risks reduction, expressed as extension in life expectancy, is addressed by Alberini *et al.* [38], Desaigues *et al.* [39] and Chilton *et al.* [40]. Finally, Aunan *et al.* [30] implement a cost-benefit analysis to estimate the net benefits of an energy saving program in Hungary that would result to significant emissions reductions. The analysis indicates that the main benefit from reduction of the concentrations of pollutants relates to improved human health. The estimated annual benefit of improved health conditions alone is likely to exceed the investments needed to implement the program even under the lowest estimates. A cost-benefit analysis is also applied by Larson *et al.* [41] to assess the efficiency of five projects leading to 25-fold reduction in mortality risk due to particulate emissions in Russia. The Value of a Statistical Life was transferred to Russia after adjustment to estimate benefits of reduced mortality. The total net present benefit of all five projects is found about $40 million which justify the undertaking of the projects on economic grounds.

### 3.2. Water Quality

Contact with unsafe drinking or bathing water can impose serious risks (both acute and delayed) to human health [42,43]. Microbe contamination of groundwater due to sewage outfalls and high concentration of nutrients in marine and coastal waters due to agricultural runoff are among the most serious threats [44]. According to the European Commission’s (EC) recent statistics, 20 percent of all surface water in the EU is seriously threatened by pollution [45]. In the infrastructurally disadvantaged developing world the water contamination problem is even more prominent [46].

Although epidemiological studies have provided evidence of severe morbidity attributed to polluted water the issue has received limited attention in terms of valuation studies. Only few studies explicitly address health effects of drinking and bathing water quality to inform efficient water resources management policies mainly in high income countries.

The health risks involved in bathing in polluted sea water are explicitly accounted in the study of Machato and Murato [47], who employed stated preference techniques to evaluate the multiple benefits of improving the quality of marine recreational waters on the Estoril coast in Portugal. Based on evidence from existing epidemiological dose-response functions a contingent valuation survey was employed to allow for a direct estimate of the health benefits of reduced water pollution. Results indicate that health risk reductions are only a small fraction of the total social benefits of water quality improvements. The sample mean WTP to avoid gastroenteritis was found to be € 55.56. Bathing water quality related health benefits are also studied by Johnson *et al.* [48], who adopted a benefit-transfer approach to evaluate health benefits associated with improved bathing water quality in Scotland. A dose-response function between the concentration of Intestinal Enterococci in bathing water and the probability of contracting gastro-enteritis was first determined and then the annual benefits of illness risk reduction were estimated on the WTP values from a stated preference study in England. Health benefits from a reduction in the risk of illness resulting from swimming in contaminated waters were found to be € 348.000 annually. Georgiou *et al.* [49] conducted a cost-benefit analysis to inform policy-makers in UK on the efficiency of the proposed measures to revise seawater quality standards set by the 1976 EC Bathing Water Quality Directive. Benefits were estimated based on data from a contingent valuation study and were then related to their costs. Results indicate that mean WTP amounts, representing the economic benefits of the revision are of the same order of magnitude as the estimated potential cost increases in average annual household water bills necessary to implement the revision.

Deviating from the contingent valuation framework, Dwight *et al.* [43] apply the cost of illness approach and Shuval [50] calculate the disability-adjusted life years (DALY), to quantify the health burden from illnesses associated with exposure to polluted recreational coastal waters. In the former study, health data on illness-related lost activity days and medical care use were used and the economic burden per gastrointestinal illness was estimated at € 31.9, the burden per acute respiratory disease at € 66.94, the burden per ear ailment at € 32.95, and the burden per eye ailment at € 23.81. In the later, the total estimated impact of the human disease attributable to marine pollution by sewage is about three million DALY per year, with an estimated economic loss of some11.16 billion euros per year.

In the developing world, health damages from drinking water contamination are examined by Dasgupta [46] and Maddison *et al.* [51] The former study estimates a health production function to derive the total cost of illness related to Diarrhoeal diseases in urban India,. Annual health costs are calculated and aggregated over the whole population are found to equal € 2,821,587. The latter estimates aggregate willingness to pay to avoid health risks, including various cancers, associated with consumption of arsenic contaminated groundwater in Bangladesh. Based on Value of Statistical Life estimation from studies in India, authors report an aggregate WTP of $2.7 billion annually to avoid mortality and morbidity cases.

### 3.3. Climate Change

An understanding of the likely impacts of climate change on human welfare is crucial for making an informed decision about the best response strategy to the enhanced greenhouse effect. Consequently, a number of studies have attempted the evaluation of climate change-related health hazards.

Bell *et al.* [17] review the literature on valuation studies assessing health consequences from greenhouse gases. Results from multiple studies provide strong evidence that the public health benefits related to greenhouse gases mitigation strategies are substantial. The review, however, is restricted to health benefits from air pollution exposure. Benefits from greenhouse gases mitigation policies are also addressed by Burtraw *et al.* [52]. Authors examine the US electricity sector and value changes to human health resulting from carbon emissions based on concentration response functions. Results indicate health-related ancillary benefits from further reductions in carbon emissions under a € 23.15 carbon tax to be about € 7.41 per metric ton of carbon reduced in the year 2010.

A review of the literature evaluating the welfare impacts of climate change, including climate variation-related diseases is also presented in Tol [5]. However the studies included provide a total cost estimation of the climate change in $ per tonne of carbon and health effects are not distinguished. Based on the existing literature, Tol concludes that policy response to climate change should be dominated by adaptation, not by mitigation.

Welfare losses associated with health impacts induced by global warming are also estimated by Bosello *et al.* [9]. Authors apply a general equilibrium macroeconomic model to infer costs estimates relating to cardiovascular and respiratory disorders, diarrhoea, malaria, dengue fever and schistosomiasis occurrences through changes in labour productivity and demand for health care. Consistent with the literature, results imply the welfare costs (or benefits) of health impacts contribute substantially to the total costs of climate change both in terms of GDP and investment.

Bateman *et al.* [53] apply a contingent valuation study to assess WTP for reductions in the skin cancer risks associated with exposure to solar UV radiation. A common valuation scenario was applied to four countries (New Zealand, Scotland, England, and Portugal) across which objectively measured risk levels, for example cancer rates, vary substantially. Authors intended to examine whether scientifically established health risks are reflected in WTP for risk reductions in these countries and results confirm that differences in stated WTP between countries reflects the variation in risk levels between those countries.

Health effects from illnesses associated with climate change are also examined in the developing world by Tseng *et al.* [54] using the dengue fever in Taiwan as a case study. The relationship between climate conditions and the number of people infected by dengue fever was first established and the monetary assessment was then attempted applying a contingent valuation study. Results indicate that people would pay € 15.78, € 70.35 and € 111.62 per year in order to reduce the probabilities of dengue fever inflection by 12%, 43%, and 87%, respectively.

## 4. The Use of Valuation Results in Policy Design

Climate change and anthropogenic forcing threaten environmental stability and with it ecosystems’ capacity to provide goods and services that can be translated to economic benefits for humans including values associated with health quality and death mitigation. Although environmental goods and services have value to society, are often neglected in policy-making as they are not traded in markets and as such are not priced. A primary cause for environmental degradation and consequent health hazards is failure to identify and internalize in decision-making the economic value of ecosystems. Given the public nature of the environmental resources, market data, if available at all, can lead to misleading decisions regarding the significance of resources protection resulting in further resources depletion and degradation. Therefore economic valuation is extremely crucial to provide the correct economic indicators and signals for the design of efficient and sustainable economic policies.

In the absence of markets, valuation studies can provide policy-makers with the necessary information to acknowledge the contribution of health benefits in the social welfare associated with environmental resources justifying the need for policy intervention to eliminate health effects from environmental hazards. Further, preference elicitation for different socio-economic groups and knowledge of the marginal valuation each group attaches to environmental improvements through valuation studies allows for equity considerations to be taken into account in the formulation of policy responses.

Once aggregated over the full range of beneficiaries, monetary benefits estimated through valuation studies can be compared with the costs of the relevant environmental or health intervention policies through cost-benefit analysis to derive useful information on the efficiency of the planned policy. Welfare changes from alternative policy initiatives can be also assessed and the impact of social, economic and attitudinal characteristics on individual valuation can be examined. In this respect, valuation studies are significant for policy-making to guide the selection of economic instruments to allocate resources among socially valuable endeavours [55].

Economic instruments should provide the necessary incentives to all different stakeholders to act in a sustainable way. To halt environmental degradation and associated health effects economic instruments should intend to provide incentives for adopting preventative measures and refraining from polluting activities. Instruments for natural resources management include standards and quotas, abstraction and pollution taxes, subsidies and tradable permits. Taxes, subsidies and quotas are fiscal policy instruments that can internalize the external costs created by natural resources use and if set at the social optimal level can ensure full cost pricing of the environmental goods and services, a necessary condition for sustainability. Tradable permits systems have been implemented in a number of countries for several pollutants and are also intoduced by the Kyoto protocol with the intention of reducing the greenhouse gases emissions in the contracting counties. Under tradable emission permits, a market for environmental quality is created in which the right to use the environment as a waste sink is priced, and traded [56]. Further liability systems (legal liability, non-compliance charges) intending to internalize and recover the costs of environmental damage through legal action causes can be established. All instruments should be consistent with the ‘polluter pays principle’ which ensures that the cost of environmental pollution is charged to users and should intend full cost recovery of the environmental damage. Distributional, environmental and sustainability effects of the implementation of each instrument should also be considered and valuation studies can be really informative in this respect. This is particularly valid for the the developing countries where decision makers are faced with the challenge of mitigating environmental risks while supporting economic growth. To ensure environmental protection while enhancing economic development, economic instruments should be properly designed and implemented and in this respect information from valuation studies is crucial.

Information from valuation studies can also assist the design of efficient insurance programs to mitigate health effects resulting from environmental stresses. Knowledge of social perception of the effects of health risks is crucial for the formulation of optimal risk mitigation/hedging strategies. These strategies should be able to allocate the aggregate social health risk between socio-economic groups in order to provide efficient, equitable and sustainable coverage against environmental health hazards.

## 5. Concluding Remarks

Environmental degradation poses a significant threat to human health worldwide. Harmful consequences of this degradation to human health are already being felt and could grow significantly worse over the next 50 years [2]. Because environment and health are so intimately linked, so too should be environmental and health policies. However, health impacts are non-marketed and thus hard to quantify in monetary terms. The subsequent risk of being ignored in policy-making is a major concern worldwide. To address this challenge a number of valuation studies have been conducted in both developing and developed countries applying different methods to capture health benefits from improved environmental quality. Valuation results are crucial for the formulation of economic instruments to internalize the externalities created by the public nature of environmental resources. The application of fiscal instruments, the introduction of charge systems and/or the creation of emission markets can only promote sustainable outcomes if set at a social optimal level. Elicitations of the preferences and valuations of different social groups through valuations is therefore essential. This paper reviews the main literature in the field. Although not exhaustive, applied research cited in this review provides substantial evidence of strong correlation between exposure to environmental hazards and health risks and reveals that there are significant values associated with longevity and health quality in both developed and developing world justifying the need for policy interventions.

Enhancing air quality and securing adequate supplies of safe drinking water is associated with significant benefits for human health and well-being. Significant benefits are also found to be associated with bathing water quality socially justifying the costs for abatement policies. Climate change effects mitigation is also of great importance in terms of public health benefits. However, certain limitations of the existing literature have been identified.

Pearce [12] argued that a major weakness of the air pollution damage literature has been the focus on outdoor pollution. Still, remarkably few studies have measured indoor air pollution which could be the focus of future research. It is also noteworthy that only contingent valuation studies have been conducted when stated preference techniques are applied to elicit public preferences for improved air quality. However the Contingent Valuation method is found to be associated with several biases (strategic bias, yes-saying bias and embedding effect among others) and thus the Choice Experiment method could provide more reliable results [57]. Future valuation efforts could therefore apply this relatively new stated preference method to assess the social benefit associated with policies attempting to improve air quality. Finally there are considerably few valuation studies on environmental health risks of air pollution in Europe.

Regarding health hazards relating to water, although an international consensus has emerged in policy regarding water quality based on growing concern on environmental and health issues there are few valuation studies eliciting public preferences for improved water quality and subsequently reduced illness risk. The need for economic analysis is, however, highly acknowledged as explicitly manifested in the recently adopted EU Water Framework Directive (2000/60/EC) [58] which calls for the application of economic principles, economic methods and economic instruments for achieving good water status for all EU waters in the most effective manner [59,60]. Given European and international calls for sustainable water resources management, authors believe that valuing health benefits from surface and groundwater water quality improvements could be a challenging direction for future research especially in the developing world where water quality issues are particularly prominent and the lack of valuations studies is noteworthy.

Moreover, to provide accurate monetary estimates of the benefits of reduced health symptoms associated with environmental hazards, collaboration between economists and epidemiologists should be further enhanced to establish more informed dose-response functions and accordingly formulate the valuation scenarios. Finally, since health benefits from environmental improvements accrue in the long run their assessment should recognize their long-run nature. It follows that discounting and the subsequent selection of a social discount rate to discount future benefits from a policy intervention is crucial to determine whether a policy passes a cost-benefit analysis test taking sustainability and inter-generational equity into consideration [61].

## Figures and Tables

**Table 1.** The 10 leading causes of death by broad income group (2004).

***Low-income countries***	***Deaths in millions***	***% of deaths***

Lower respiratory infections	2.94	11.2
Coronary heart disease	2.47	9.4

**Diarrhoeal diseases**	1.81	6.9

*HIV/AIDS*	*1.51*	*5.7*

Stroke and other cerebrovascular diseases	1.48	5.6
Chronic obstructive pulmonary disease	0.94	3.6
Tuberculosis	0.91	3.5
Neonatal infections	0.90	3.4
Malaria	0.86	3.3

**Prematurity and low birth weight**	0.84	3.2
***High-income countries***	***Deaths in millions***	***% of deaths***

Coronary heart disease	1.33	16.3
Stroke and other cerebrovascular diseases	0.76	9.3
Trachea, bronchus, lung cancers	0.48	5.9
Lower respiratory infections	0.31	3.8
Chronic obstructive pulmonary disease	0.29	3.5
Alzheimer and other dementias	0.28	3.4
Colon and rectum cancers	0.27	3.3
Diabetes mellitus	0.22	2.8
Breast cancer	0.16	2.0

**Stomach cancer**	0.14	1.8

Source: World Health Organization [10].

## Appendix

**Table:** Summary of Valuation Studies

**Author**	**Study Area**	**Valuation Technique**	**Environmental hazard**	**Results**

Brajer *et al.* 2006 [32]	Hong-Kong	Dose –response function/benefits transfer	Air pollution	Authors find that there remain significant health gains, ranging between €1.4 billion and € 4.6 billion over the period 2003–2012 that could be achieved should Hong Kong further reduce ambient pollution levels.
Mead and Brajer 2006 [33]	China	Dose –response function/benefits transfer	Air pollution	Authors report a total valuation of over €9.9 billion for a program that would result in nearly a billion morbidity instances avoidance.
Li *et al.* 2003 [29]	Shanghai	Benefit-Cost ratio	Air pollution	The study shows that the benefit-to-cost ratio is in the range of 1 to 5 for the power-sector initiative and 2 to 15 for the industrial-sector initiative. Thus, there appear to be substantial benefits associated with air pollution control in developing cities.
Aunan *et al.* 1998 [30]	Hungary	Cost-Benefit Analysis	Air pollution	The estimated annual benefit of improved health conditions alone is likely to exceed the investments needed to implement the program even under the lowest estimates.
Miraglia 2007 [31]	Brasil	Cost-Benefit Analysis	Air pollution	Estimated benefits using an averted behaviour technique far outweighed measured costs indicating that Sao Paulo would benefit from the biodiesel use.
Wang and Mullahy 2006 [25]	China	Contingent Valuation	Air pollution	Authors report that respondents are on average willing to pay WTP of € 28.7 for a program that would cut one quarter of premature deaths due to air pollution.
Wang and Zhang 2009 [26]	China	Contingent Valuation	Air pollution	The mean WTP was estimated to be €10.79 per person per year
Chau *et al.* 2007 [35]		Revealed and Stated Preference techniques/Meta-analysis	Air pollution	Findings indicate that there would be some benefit gains for the owners-employers and the society if certain regular filter sets were adopted.
Gupta 2008 [34]	India	Cost of illness	Air pollution	Results indicate that the mean worker from Kanpur would gain €2.61 per year if air pollution were reduced to a safe level.
Hammit and Zhou 2006 [27]	China	Contingent Valuation	Air pollution	The sample average median WTP to prevent an episode of cold ranges between € 2.5 and € 4.99 while the WTP to prevent a statistical case of chronic bronchitis ranges between € 416 and € 832.73.
Alberini *et al.* 1997 [28]	Taiwan	Contingent Valuation	Air pollution	Median WTP to avoid a recurrence of the average episode is found to be € 41.35.
Navrud 2001 [37]	Norway	Contingent Valuation	Air pollution	Mean WTP for an environmental program that would result to reduced health risks (avoiding one additional day of the health symptoms) ranges from €16.62 for coughing to €44.2 for the shortness of breath.
Alberini *et al.* 2006 [38]	UK, France and Italy	Contingent Valuation	Air pollution	Mean WTP from the pooled sample is €1168 per year for a 5 to 1000 mortality risk reduction while the value of a loss of one year’s life expectancy is between €56,903 and €146,913.
Desaigues *et al*. 2003 [39]	France	Contingent Valuation	Air pollution	Mean WTP for a mortality risk reduction of 1 to 1000 between the age of 70 and 80 is € 458.6.
Chilton *et al.* 2004 [40]	UK	Contingent Valuation	Air pollution	Mean annual WTP is € 138.82 for the one month life expectancy extension sample, € 157.31 for the three months sample, and € 187.38 for the six months sample.
Larson *et al.* 1999 [41]	Russia	Value of a Statistical Life/Cost-Benefit Analysis	Air pollution	The total net present benefit of five projects to reduce particulate emissions is estimated at about € 37.23 million
Machato and Murato 2002 [47]	Portugal	Contingent Valuation/Contingent ranking	Bathing water pollution	The sample mean WTP to avoid gastroenteritis episodes was found to be €55.56.
Johnson *et al.* 2008 [48]	Scotland	Dose response function/Benefit Transfer	Bathing water pollution	Health benefits from a reduction in the risk of illness resulting from swimming in contaminated waters were found to be €348.000 annually.
Georgiou *et al.* 2000 [49]	UK	Cost-Benefit Analysis	Bathing water pollution	Results indicate that mean WTP amounts, representing the economic benefits of the revision are of the same order of magnitude as the estimated potential cost increases in average annual household water bills necessary to implement the revision.
Dasgupta 2004 [46]	India	Cost of illness	Drinking water pollution	Annual health costs related to Diarrhoeal diseases are aggregated to the whole population are found to equal € 34.19.
Dwight *et al.* 2005 [43]	US	Cost of illness	Bathing water pollution	The economic burden per gastrointestinal illness was estimated at € 31.9, the burden per acute respiratory disease at € 66.94, the burden per ear ailment at € 32.95, and the burden per eye ailment at € 23.81.
Shuval 2003 [50]	World	Disability-Adjusted Life Years (DALY)	Bathing water pollution	The total estimated impact of the human disease attributable to marine pollution by sewage is about 3 million DALY per year, with an estimated economic loss of some € 11.16 billion per year.
Maddison *et al.* 2005 [51]	Bangladesh	Value of Statistical Life	Groundwater contamination with Arsenic	Authors report an aggregate WTP of €2.26 billion annually to avoid mortality and morbidity cases
Burtraw *et al.* 2003 [52]	US	Carbon tax	Climate Change	Authors find health-related ancillary benefits from further reductions in carbon emissions under a € 23.15 carbon tax to be about € 7.41 per metric ton of carbon reduced in the year 2010.
Bosello *et al.* 2006 [9]	World	General equilibrium macroeconomic model	Climate Change	Results imply the welfare costs (or benefits) of health impacts contribute substantially to the total costs of climate change both in terms of GDP and investment
Bateman *et al.* 2005 [53]	Portugal, England, Scotland, New Zeland	Contingent Valuation/Natural Experiment	Climate Change	For both the private and public good, proposed to reduce health risks from exposure to solar radiation, WTP is highest in New Zealand followed by Scotland and England, with the lowest value being given by the Portuguese sample. Results suggest that WTP reflects differences in exogenous health risks in the four countries.
Tseng *et al.* 2009 [54]	Taiwan	Contingent Valuation	Climate Change	Results indicate that people would pay €15.78, € 70.35 and € 111.62 per year in order to reduce the probabilities of dengue fever inflection by 12%, 43%, and 87%, respectively.
